# SLC1A5 co-expression with TALDO1 associates with endocrine therapy failure in estrogen receptor-positive breast cancer

**DOI:** 10.1007/s10549-021-06298-1

**Published:** 2021-07-19

**Authors:** Lutfi H. Alfarsi, Rokaya El Ansari, Madeleine L. Craze, Omar J. Mohammed, Brendah K. Masisi, Ian O. Ellis, Emad A. Rakha, Andrew R. Green

**Affiliations:** 1 Nottingham Breast Cancer Research Centre, Academic Unit for Translational Medical Sciences, School of Medicine, University of Nottingham Biodiscovery Institute, University of Nottingham, University Park, Nottingham, NG7 2RD UK; 2 Cellular Pathology, Nottingham University Hospitals NHS Trust, Nottingham City Hospital, Hucknall Road, Nottingham, NG5 1PB UK

**Keywords:** SLC1A5, Tamoxifen resistance, ER, Breast cancer, TALDO1

## Abstract

**Purpose:**

Identification of effective biomarkers for the benefit of endocrine treatment and understanding the molecular pathways that contribute to the development of resistance are of crucial importance to the management of luminal breast cancer. The amino acid transporter SLC1A5 has emerging importance as a prognostic marker and potential therapeutic target in various types of cancer. This study aims to investigate its role in luminal breast cancer as a potential predictive marker for endocrine treatment.

**Methods:**

SLC1A5 expression was assessed at the transcriptomic and proteomic levels in large, well-characterized cohorts of luminal breast cancer. The sensitivity to endocrine therapy after SLC1A5 knockdown was investigated in vitro, using MCF7 and MDA-MB-175 cell lines. Bioinformatic analyses were performed to study the interacting networks of SLC1A5 and to identify a key co-expressed gene with SLC1A5.

**Results:**

Here, we showed that patients with tumors that highly expressed SLC1A5 associated with a high risk of relapse after endocrine treatment. In vitro, depletion of SLC1A5 increases the sensitivity of luminal breast cancer cells to tamoxifen. TALDO1 was identified as key co-expressed gene with SLC1A5, and in vitro knockdown of SLC1A5 showed reduction in TALDO1 expression. Indeed, TALDO1 was associated with poor clinical outcomes in patients who were subject to endocrine therapy.

**Conclusion:**

These findings suggest that metabolic alterations, particularly the interaction between the key amino acid transporter SLC1A5 and metabolic enzyme TALDO1, could affect the sensitivity of endocrine therapy. This study demonstrated the prognostic value of both SLC1A5 and TALDO1 as biomarkers in luminal breast cancer.

**Supplementary Information:**

The online version contains supplementary material available at 10.1007/s10549-021-06298-1.

## Introduction

Estrogen receptor (ER) is considered a driving transcription factor in breast cancer cells, and up to 70% of breast cancer patients are ER-positive (ER +), luminal tumors. Endocrine therapy has been widely used in clinical practice as an adjuvant treatment for breast cancer. However, despite its undisputed efficacy, up to one-third of patients will eventually become resistant to this treatment, leading to tumor recurrence and metastasis [1, 2]. The identification of biomarkers to predict endocrine therapy benefit in addition to ER status is therefore of crucial importance in stratifying luminal patients for targeted therapy.

Dysregulated metabolic pathways are observed generally across many types of cancer cells and are considered hallmarks of cancer, where they are able to regulate their metabolism to sustain rapid proliferation [3]. The rapid growth of cancer cells requires the maintenance of amino acids for protein synthesis and metabolic demands. Glutamine is the second primary metabolite to nourish cancer cell proliferation. Given the increasing demand of glutamine in malignant tumors, some tumors cannot survive in the absence of exogenous glutamine, known as glutamine addiction [4]. Targeting the key components of glutamine metabolism to prevent glutamine uptake provide potential targets to block tumor growth.

SLC1A5, also known as ASCT2, is a sodium-dependent transporter which regulates the influx of neutral amino acids including glutamine [5, 6]. SLC1A5 has a key role in sustaining glutamine metabolism and tumor growth and progression [7]. Several studies demonstrate that the knockdown of SLC1A5 prevents glutamine uptake which in turn inhibits cell proliferation, indicating the importance of this solute carrier during tumorigenesis in different types of cancer [7–12].

In breast cancer, high expression of SLC1A5 mediates glutamine uptake and sustained cell proliferation [13]. However, no previous study has reported utility of SLC1A5 in predicting the benefit of endocrine therapy in luminal breast cancer. Therefore, in this study, we investigated the prognostic role of SLC1A5 in luminal breast cancer and determined the effect of SLC1A5 silencing as to the sensitivity of endocrine treatment. Finally, we performed bioinformatics analysis to identify associated networks with SLC1A5 which could have potential roles in response to endocrine treatment in luminal breast cancer.

## Materials and methods

### *mRNA* analysis

To investigate the prognostic value of *SLC1A5* and *TALDO1 mRNA* expression in luminal breast cancer patients and its role as predictive markers of clinical outcome for patients who were subject to endocrine treatment, three publicly available transcriptomic datasets were used. The METABRIC [14] was used as a discovery cohort. The characteristics of this cohort are summarized in (Supplementary Table 1). In the METABRIC cohort, DNA/RNA was extracted from primary tumor samples and were hybridized using the Illumina Human HT-12 v3 platforms. Data were pre-processed and normalized as described previously [14]. In this cohort, patients who were ER + and/or lymph node negative did not receive adjuvant chemotherapy.

The Kaplan Meier Plotter-Breast Cancer (KM-Plotter) dataset [15], was used as a validation cohort for the prognostic and predictive value of *SLC1A5* and *TALDO1 mRNA* expression in luminal breast cancer. Further, we used Breast Cancer Gene-Expression Miner v4.3 (bc-GenExMiner v4.3) [16] to analyze the prognostic value of *TALDO1* on luminal breast cancer and also test the correlation of *SLC1A5* or *TALDO1* with other genes.

### Analysis of protein expression

Investigation of SLC1A5 and TALDO1 protein expression was assessed in well-characterized series of luminal invasive breast cancer patients, with long-term follow-up, who were diagnosed at Nottingham City Hospital between 1989 and 2006. Patient management was uniform and based on tumor characteristics by NPI and hormone receptor status. No adjuvant therapy was given to patients with good prognostic NPI scores (≤ 3.4), while for patients with poor NPI scores (> 3.4) endocrine therapy was given. Premenopausal patients within the moderate and poor prognostic NPI were given chemotherapy, whereas postmenopausal patients with moderate or poor NPI were candidates for hormonal therapy. None of the patients in this study received neoadjuvant therapy. The characteristics of this cohort are summarized in (Supplementary Table 1).

Protein expression of SLC1A5 and TALDO1 was assessed using immunohistochemistry (IHC) on 4-μm tissue microarray sections using Novolink polymer detection system (RE7150-K, Leica Biosystems, UK), as previously described [17]. Sections with SLC1A5 antibody (1:200; HPA035240, Sigma-Aldrich, UK) were incubated for 30 min at room temperature, while sections with TALDO1 antibody (1:500; GR113889, Abcam, UK) were incubated for 1 h at room temperature. Slides were digitalized and analyzed using high-resolution digital images (Nanozoomer, Hamamatsu Photonics, UK) and viewing software (Xplore; Philips, UK). Evaluation of SLC1A5 and TALDO1 staining in invasive tumor cells was performed using the modified histochemical score (H-score) [18]. TMA cores were only assessed if the invasive tumor burden was > 15%.

### Biological co-expression networks and pathway analysis

The Tissue and Cancer Specific Biological Networks (TCSBN) [19] was used to explore the biological networks of SLC1A5 in breast cancer to identify genes that directly interacted or co-expressed with SLC1A5. The interacting network was drawn by Cytoscape version 3.6.1 [20] using the top-25 interacted genes with SLC1A5. Next, Gene Ontology analysis was performed to determine the most enriched categories of biological processes for the identified 25 genes co-expressed with SLC1A5. Kyoto Encyclopedia of Genes and Genomes (KEGG) pathway analysis was then used to functionally interpret the 25 genes derived from the co-expression network analysis. Webgestalt tool was used to perform the Gene Ontology enrichment and KEGG pathway analysis [21].

### Cell lines and reagents

Luminal breast cancer cell lines MCF7 and MDA-MB-175 were obtained from American Type Culture Collection (Rockville, MD, USA). MCF7 cells were cultured in RPMI-1640, while MDA-MB-175 cells were maintained in Dulbecco’s Modified Eagle medium both supplemented with 10% fetal bovine serum. Both cell lines were incubated at 37 °C with 5% CO_2_. All experiments were performed with mycoplasma-free cells. All the cell lines have been authenticated using Short Tandem Repeat (STR) profiling within the last three years. The tamoxifen active metabolite 4-Hydroxytamoxifen treatment was purchased from Sigma-Aldrich (SML1666). siRNA-targeting SLC1A5 (ID: s12918) along with a scrambled negative siRNA control (4,390,843) were synthesized by Ambion, ThermoFisher.

### siRNA knockdown of SLC1A5

MCF7 and MDA-MB-175 cells were transfected with siRNA-targeting SLC1A5 alongside a scrambled negative siRNA control according to the manufacturer’s protocol. Cells were seeded into T-25 flasks at a density of 500,000 cells. After 24 h, cells were transfected with either scrambled negative control siRNA or 5 nM siRNA against SLC1A5 using Lipofectamine RNAiMAX (Invitrogen). Transfected cells were either trypsinized and seeded in 96-well plates the following day for tamoxifen sensitivity assay, or incubated for five days which then lysed with lysis buffer for western blot.

### Tamoxifen sensitivity assay

The 3-(4,5-dimethyl-2-yl)-5-(3-carboxymethoxyphenyl)-2-(4-sulfophenyl)-2H-tetrazolium, inner salt; MTS (CellTiter 96 AQueous One Solution Cell Proliferation Assay, Promega) assay was used according to manufacturer’s instructions. Briefly, siRNA control cells or after 24 h SLC1A5 siRNA transfection, cells were seeded in 96-well plates at a density of 2000 cells/well and allowed to adhere for 24 h. The medium was replaced with fresh medium with increasing concentrations of 4-hydroxytamoxifen (2–15 μM) and incubated for 72 h. 20 μl of MTS reagent was added to each well and incubated for 1 h before the absorbance was measured at 492 nm using Infinite F50 (Tecan, UK).

### Western blot analysis

Cells were lysed with lysis buffer containing RIPA buffer (ThermoFisher Scientific), mini protease inhibitor cocktail complete (Roche) and phosphatase inhibitor (Sigma). Samples were loaded on 12% SDS–polyacrylamide gel electrophoresis (PAGE) gels and transferred to PVDF membranes (Immobilon-FL) or nitrocellulose membranes (GE Healthcare). Membranes were incubated overnight with the following primary antibodies: anti-SLC1A5 (1:150; HPA035240, Sigma-Aldrich, UK), anti-TALDO1 (1:500; GR113889, Abcam, UK) and Anti-β-actin (1:5000; Sigma-Aldrich, UK) as a loading control. The analysis and quantification of immunoblotting were performed using the Odyssey Fc with Image Studio 4.0 (LI-COR Biosciences), as previously described [17].

### Statistical analysis

SPSS statistical software (version 25, Chicago, IL, USA) was used for data analysis. The Chi-square test was performed for inter-relationships between categorical variables, while for continuous variables Spearman’s correlation coefficient test was used. Kaplan–Meier survival curves were used to assess the association of SLC1A5, TALDO1 or SLC1A5/TALDO1 co-expression with clinical outcome. Cox regression analysis was used to evaluate the independent prognostic significance of SLC1A5, TALDO1 and SLC1A5/TALDO1 co-expression. Benjamini–Hochberg procedure for multiple test correction was performed. The dichotomization of SLC1A5 and TALDO1 *mRNA* and protein expression into low and high groups was determined using X-Tile (X-Tile Bioinformatics Software, Yale University, version 3.6.1) based on prediction of outcome. Student’s t-tests using PRISM were performed to determine the effects of SLC1A5 knockdown and tamoxifen treatment. Data are represented as the mean ± standard deviation (SD) of at least two independent experiments performed in triplicate. All statistical tests were two-sided, and *P* values < 0.05 were considered significant.

## Results

### Clinical significance of SLC1A5

**Fig. 1:**
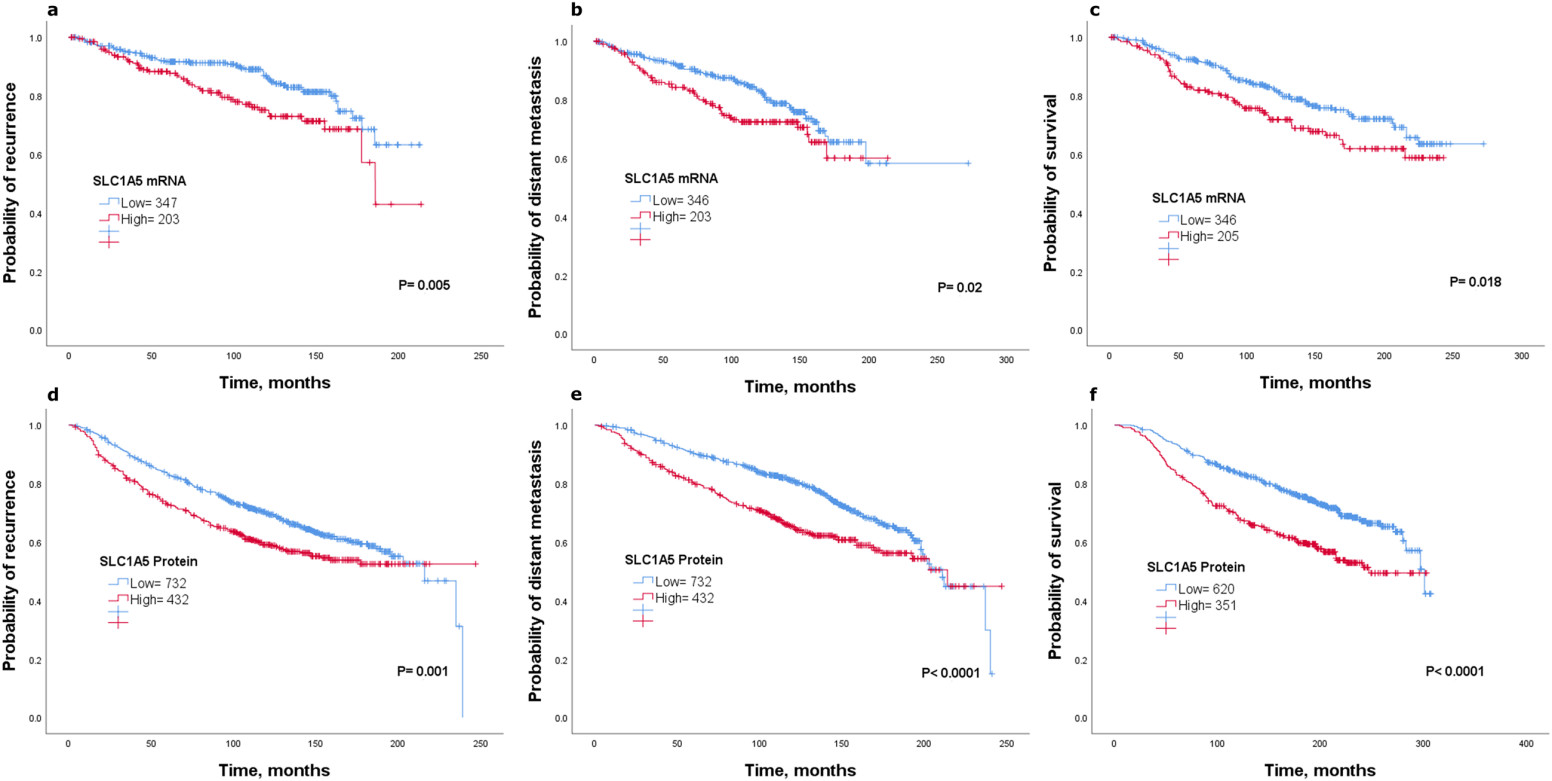
Kaplan–Meier of *SLC1A5 mRNA* and patient outcome in luminal breast cancer using the METABRIC cohort** a** recurrence**, b** distant metastasis, and** c** survival. Kaplan–Meier of SLC1A5 protein and patient outcome in luminal breast cancer **d** recurrence, **e** distant metastasis, and **f** survival

We first investigated the prognostic value of *SLC1A5 mRNA* expression in luminal breast cancer patients using METABRIC and KM-Plotter datasets. Patients with higher *SLC1A5* tumor levels in METABRIC had an unfavorable outcome compared with those patients with low SLC1A5 levels (*P* < 0.05; Fig. 1a–c). Consistent with this finding, high *SLC1A5 mRNA* expression was significantly associated with a high risk of recurrence, distant metastasis and death from breast cancer using KM-Plotter (*P* < 0.05; Supplementary Fig. 1a–c).

Next, we performed IHC staining on a large series of samples (*n* = 1188) for patients with luminal breast cancer to analyze the protein expression of SLC1A5 with clinicopathological features and clinical outcome. The expression of SLC1A5 was mainly observed in the membrane of the invasive breast cancer cells, with expression levels varying from negative to high (Supplementary Fig. 1d, e). High expression of SLC1A5 was associated with aggressive clinicopathological parameters including larger tumor size, poor Nottingham Prognostic Index (NPI), high tumor grade, vascular invasion, and high Ki67 expression (A cut-off point of 10 was used to define its positivity) (*P* < 0.05), Table 1. In terms of clinical outcome, patients were divided into two groups according to SLC1A5 expression (low vs high). Kaplan–Meier survival analysis was performed, and protein results were consistent with the *SLC1A5* transcript whereby patients with higher SLC1A5 expression had adverse outcome compared to patients with low SLC1A5 expression (Fig. 1d–f). Taken together, these results suggest increased SLC1A5 transcription and translation associates with aggressive features in luminal breast cancer leading to cancer progression and decreased survival.

**Table 1:** Association of SLC1A5 protein expression and clinicopathological parameters in luminal breast cancer

	SLC1A5 expression		*P*	P*
	Low No. (%)	High No. (%)		
Age			0.02	0.03
< 50	244 (57.3)	182 (42.7)		
≥ 50	598 (63.6)	342 (36.4)		
Tumor size (cm)			0.002	0.003
< 2 cm	490 (65.4)	259 (34.6)		
≥ 2 cm	352 (57.1)	265 (42.9)		
Tumor grade			3.97e – 22	< 0.0001
1	248 (78.7)	67 (21.3)		
2	377 (65.9)	195 (34.1)		
3	214 (45.1)	261 (54.9)		
NPI			5.0e – 13	< 0.0001
GPG	404 (73.1)	149 (26.9)		
MPG	357 (56)	281 (44)		
PPG	81 (46.3)	94 (53.7)		
Nodal stage			0.007	0.01
1	556 (64.8)	302 (35.2)		
2	229 (56.3)	178 (43.7)		
3	54 (55.7)	43 (44.3)		
Vascular invasion			1.17e – 10	< 0.0001
Negative	631 (67.4)	305 (32.6)		
Positive	207 (49.1)	215 (50.9)		
Endocrine therapy			0.001	0.002
No	437 (68.5)	201 (31.5)		
Yes	307 (59.3)	211 (40.7)		
Progesterone receptor			0.12	0.13
Negative	161 (57.3)	120 (42.7)		
Positive	637 (62.3)	385 (37.7)		
Ki67			0.03	0.038
Negative	231 (65.3)	123 (34.7)		
Positive	343 (58.3)	245 (41.7)		

*GPG* Good prognostic group, *MPG* Moderate prognostic group, *PPG* Poor prognostic group

P* Adjusted *P* values

### SLC1A5 expression correlates with poor clinical outcome in endocrine-treated patients

**Fig. 2:**
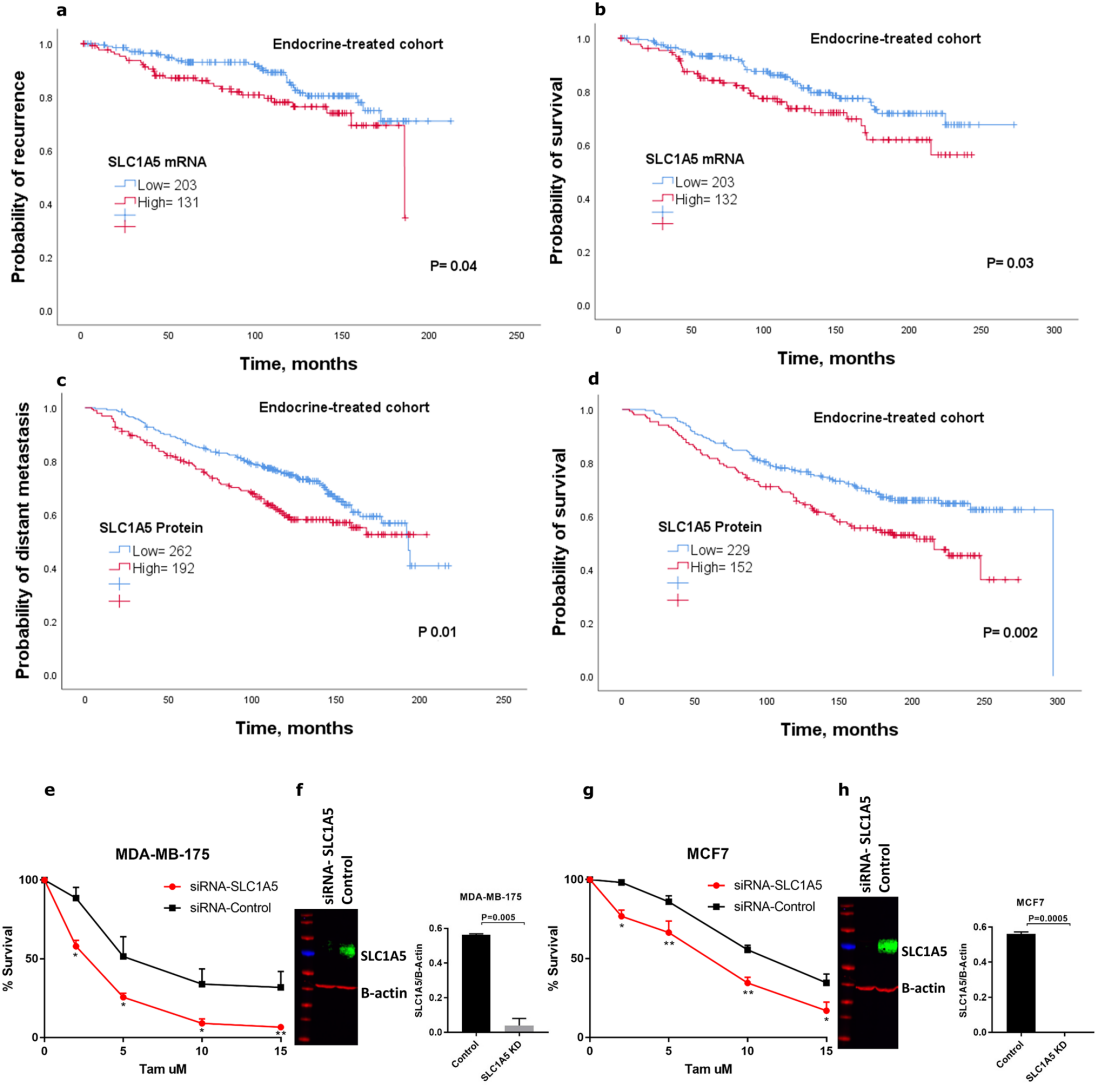
Kaplan–Meier of *SLC1A5 mRNA* expression in patients with luminal breast cancer who received endocrine treatment only using the METABRIC cohort** a** recurrence and** b** survival. Kaplan–Meier of SLC1A5 protein expression in patients with luminal breast cancer who received endocrine treatment only **c** distant metastasis and **d** survival. Tamoxifen sensitivity assay in **e** MDA-MB-175 cells and **f** efficiency of SLC1A5 knockdown, while **g** MCF7 cells and **h** efficiency of SLC1A5 knockdown. MDA-MB-175 cells and MCF7 cells were transfected with siRNA-negative control or siRNA-targeting SLC1A5 and treated with different concentrations of 4-Hydroxytamoxifen (2, 5, 10, 15 μM) for 72 h. Cell viability was measured using MTS assay. Results shown are mean ± SE of at least four independent experiments performed in triplicate. Asterisks denote *P* values as follows: *P < 0.05; **P < 0.005. The efficiency of SLC1A5 knockdown in MDA-MB-175 and MCF7 cells was confirmed by Western blotting. B-actin was used as a loading control. Results are plotted as mean ± SE from at least two independent experiments

Based on the above results, we decided to evaluate the clinical relevance of SLC1A5 with the efficacy of endocrine treatment. We analyzed the clinical outcome of patients with luminal breast cancer who received endocrine therapy alone. Analysis of the METABRIC cohort revealed that patients with tumors that highly expressed *SLC1A5 mRNA* showed significantly poorer outcomes than those with low *SLC1A5* expression (*P* < 0.05; Fig. 2a, b). This finding was further validated using KM-Plotter dataset where high *SLC1A5 mRNA* expression was significantly associated with a high risk of recurrence after endocrine treatment (*P* < 0.05; Supplementary Fig. 1f). Kaplan–Meier survival revealed that high SLC1A5 protein expression was also associated with poor clinical outcomes in patients treated with endocrine therapy (*P* < 0.05; Fig. 2c, d).

Next, to investigate the independent prognostic value of SLC1A5 protein expression we used multivariate Cox analysis. Results showed that SLC1A5 expression independently predicted poor prognosis in patients with luminal breast cancer (*P* < 0.05), (Supplementary Table 2). Further analysis on the subgroup of patients who were treated with endocrine therapy alone revealed that SLC1A5 protein expression remained a predictive marker for high risk of breast cancer death (*P* = 0.03), (Supplementary Table 2).

### SLC1A5 depletion increased sensitivity of luminal breast cancer cells to tamoxifen

To confirm that high SLC1A5 expression contributes to the poor response to endocrine therapy in the above clinical findings, we tested whether SLC1A5 knockdown increased sensitivity of breast cancer cells to tamoxifen. To do that we knocked down SLC1A5 in MCF7 and MDA-MB-175 cells using siRNA-targeting SLC1A5. Cells were treated with tamoxifen at different concentrations and the cell viability was measured. Results revealed that efficiency of tamoxifen treatment in MDA-MB-175 cells was significantly enhanced upon silencing of SLC1A5 compared with control cells (Fig. 2e). The efficiency of SLC1A5 knockdown was confirmed by western blotting (Fig. 2f). Indeed, we observe an effect on response to tamoxifen treatment after depletion of SLC1A5 in MCF7 cells as well (Fig. 2g, h). Consistent with our clinical findings, SLC1A5 knockdown resulted in increased sensitivity to tamoxifen in luminal breast cancer cells, suggesting that high SLC1A5 expression might play a crucial role in therapeutic response to endocrine treatment.

### Co-expression networks with SLC1A5

**Fig. 3:**
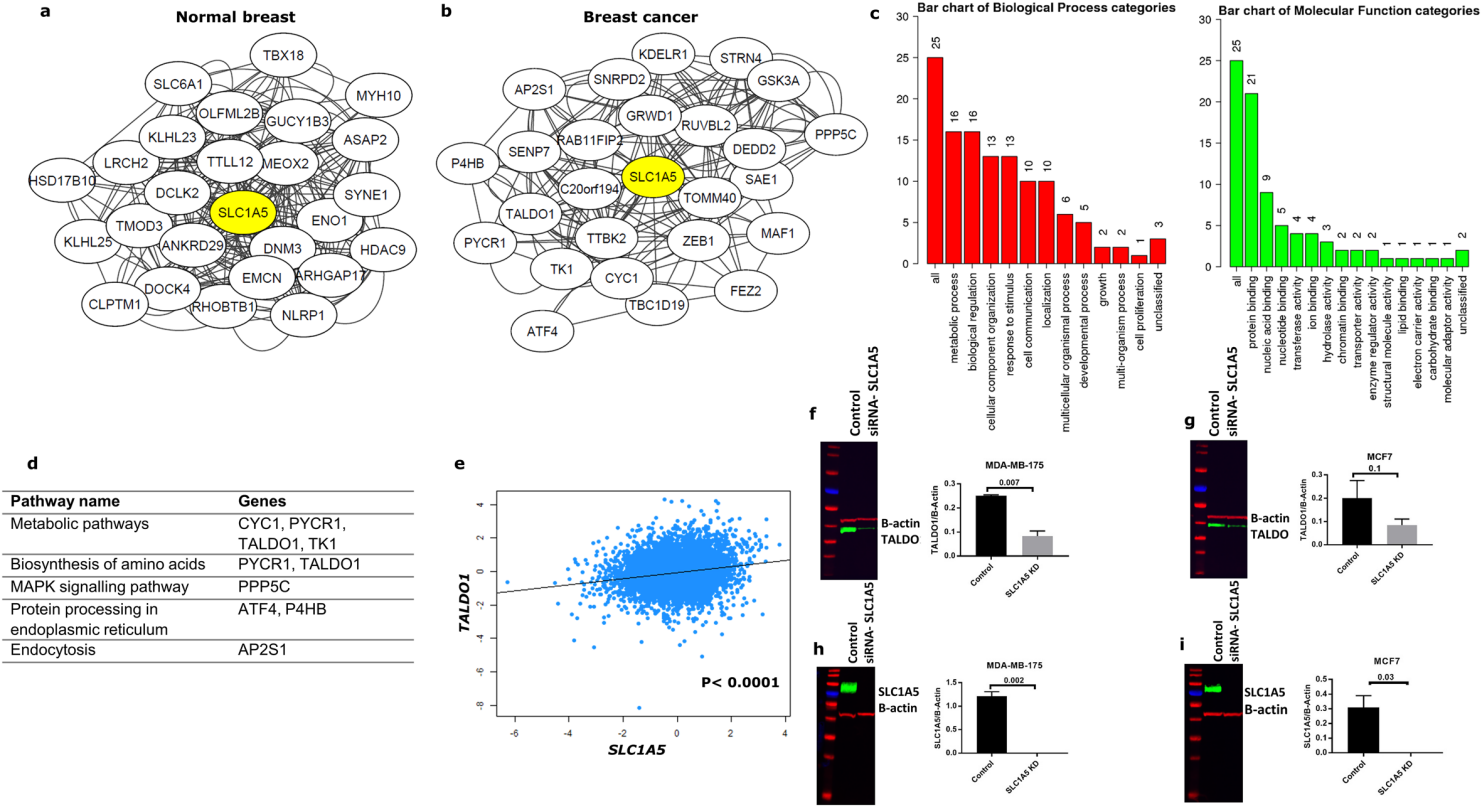
Co-expression interacting networks of genes with SLC1A5 in **a** normal breast tissue and **b** breast cancer tissue using The Tissue and Cancer Specific Biological Networks tool. **c** Results of the gene ontology analysis of enriched biological processes and molecular functions of the 25 interacted genes with SLC1A5 in breast cancer, while **d** represents the pathway analysis. **e** *SLC1A5 mRNA* correlation with *TALDO1 mRNA* using bc-GenExMiner v4.3. Effect of SLC1A5 knockdown on the expression of TALDO1 in **f** MDA-MB-175 and **g** MCF7 cells using Western blotting on the left and quantification for the protein expression of TALDO1 shown on the right. The efficiency of SLC1A5 knockdown in **h** MDA-MB-175 and **i** MCF7 cells was confirmed by Western blotting on the left, and quantification of protein shown on the right. B-actin was used as a loading control. Results are plotted as mean ± SE from at least two independent experiments

To begin investigating the biological co-expression networks associated with SLC1A5 in breast cancer, we used TCSBN database. This database allowed us to explore the relationships between genes and biological functions in breast cancer via the generation of co-expression networks in gene-centric manner (SLC1A5). We generated co-expression networks according to the top-25 genes that highly interacted with SLC1A5 in both normal breast and breast cancer tissues (Fig. 3a, b). It is obvious from the results that all genes within interacting networks in breast cancer were different from those in normal breast tissue, indicating those genes might have important roles in metabolism. The transcripts positively associated with *SLC1A5* included *SAE1, CYC1, ATF4, MAF1, P4HB, SNRPD2, TK1, PYCR1, GSK3A, DEDD2, TALDO1, TOMM40, STRN4, GRWD1, AP2S1, RUVBL2, PPP5C, and KDELR1,* whereas *FEZ2, SENP7, TTBK2, C20orf194, TBC1D19*, *RAB11FIP2*, and *ZEB1* were negatively associated.

To further explore specific functions of the identified transcripts associated with SLC1A5 in breast cancer, we performed gene ontology analysis using the Webgestalt tool. Results showed that the majority of the SLC1A5-correlated genes were significantly enriched in metabolic processes and biological regulations in terms of biological processes, whereas for molecular functions, the majority were enriched in protein binding (Fig. 3c). Next, KEGG Pathway analysis was used to identify common biological pathways and gene targets to be chosen for further investigation. The most highly enriched biological pathways were the metabolic, biosynthesis of amino acids and MAPK signaling pathways (Fig. 3d). These data suggest that increased SLC1A5 expression in luminal breast cancer potentially drives metabolic changes that affect the response to endocrine treatment.

### TALDO1 positively correlates with SLC1A5

To further narrow the scope to identify putative key genes associated with SLC1A5 in luminal breast cancer, genes within the top three pathways were selected (*TALDO1*, *CYC1*, *PYCR1*, *TK1* and *PPP5C*) for further analysis. As an initial evaluation, we performed *mRNA* analysis using bc-GenExMiner v4.3 dataset to determine the correlation of the selected genes to *SLC1A5*. Results showed that all the selected candidate genes were positively associated with *SLC1A5* in luminal breast cancer (*P* < 0.0001; Supplementary Fig. 2a). Having determined their correlation with *SLC1A5*, we next asked which of these genes have a key clinical role in luminal breast cancer and specifically in terms of benefit from endocrine treatment. In order to assess the prognostic value of each of the 5 selected genes on patients who received endocrine treatment only, Kaplan Meier analysis was performed using the METABRIC cohort. Among them, we found a significant association of only *TALDO1 mRNA* expression and worse clinical outcome in patients who were subject to endocrine therapy alone (*P* < 0.05; Supplementary Fig. 2b–f). Therefore, TALDO1 was selected for further analysis.

As we found that *TALDO1 mRNA* was positively correlated with *SLC1A5 mRNA* expression in luminal breast cancer (correlation coefficient = 0.23; *P* < 0.0001; Fig. 3e), we next tested whether depletion of SLC1A5 would affect the expression of TALDO1 in luminal breast cancer cell lines to confirm our findings from the network analysis. Interestingly, results showed that knockdown of SLC1A5 in human MDA-MB-175 cells led to a decrease in TALDO1 protein abundance (Fig. 3f). For MCF7 cells, there was also a decrease in TALDO1 expression after silencing of SLC1A5 but this did not reach significance (Fig. 3g). The efficiency of SLC1A5 knockdown was confirmed by western blotting (Fig. 3h, i). These results suggest that SLC1A5 participates in the regulation of TALDO1 expression in luminal breast cancer, and that TALDO1 might have an important functional role in luminal breast cancer.

### Clinical significance of TALDO1

**Fig. 4:**
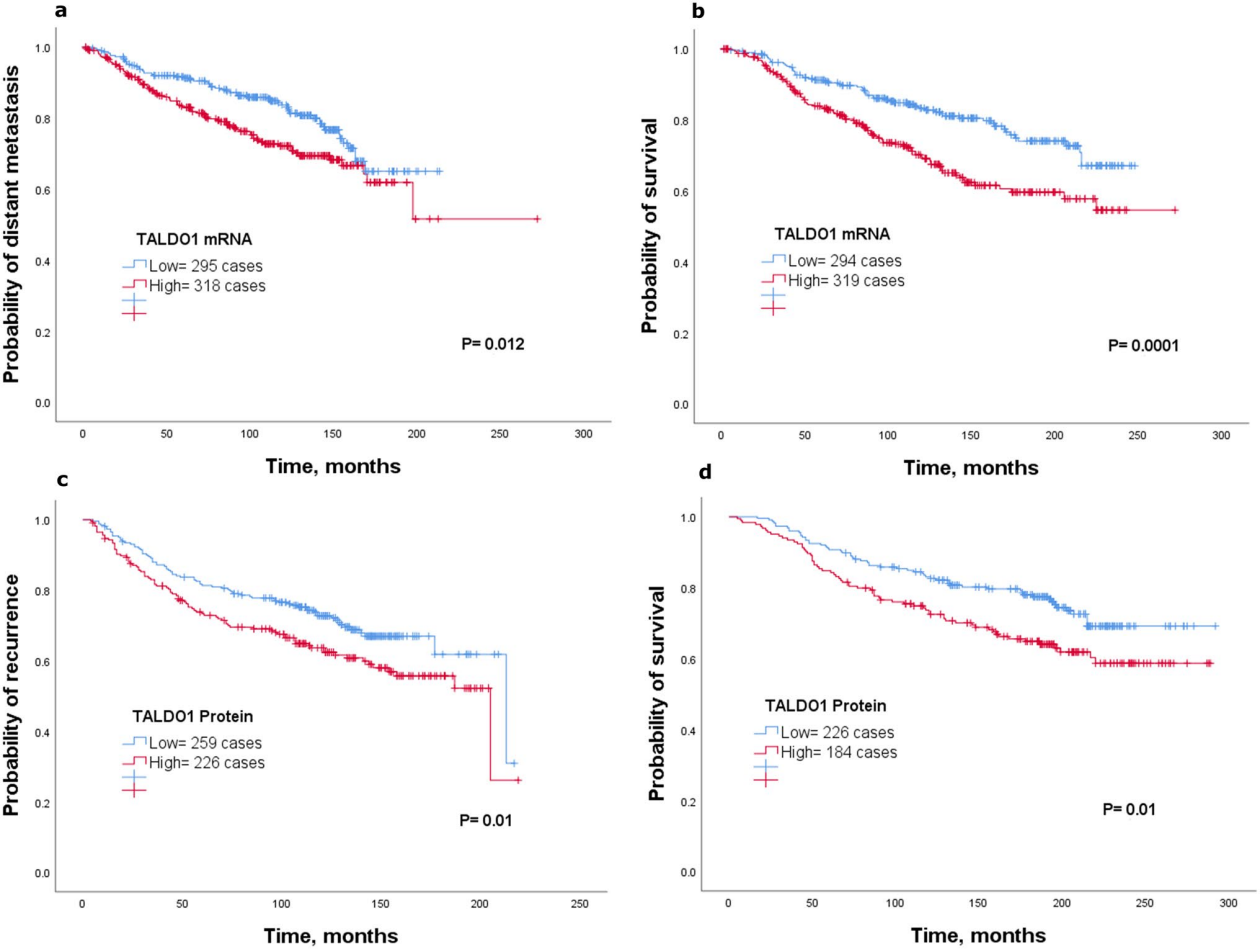
Kaplan–Meier of *TALDO1 mRNA* and patient outcome in luminal breast cancer using the METABRIC cohort **a** distant metastasis and **b** survival. Kaplan–Meier of TALDO1 protein and patient outcome in luminal breast cancer using Nottingham cohort **c** recurrence and **d** survival

On basis of the network analysis and positive correlation of *TALDO1* with *SLC1A5* in luminal breast cancer, we performed further analysis with a focus on the potential clinical role of TALDO1 in luminal breast cancer and prediction of benefit from endocrine treatment. Therefore, we studied the expression of *TALDO1 mRNA* with clinical outcome on luminal breast cancer samples using the METABRIC, KM-Plotter and bc-GenExMiner v4.3 datasets. Our results found that high *TALDO1 mRNA* expression was significantly associated with a high risk of distant metastasis and death from breast cancer (*P* < 0.05; Fig. 4a, b) in the METABRIC cohort. These results were further validated using KM-Plotter, where high expression of *TALDO1 mRNA* was associated with a high probability of recurrence, distant metastasis and death from breast cancer (*P* < 0.05; Supplementary Fig. 3a–c). A similar result was found using bc-GenExMiner v4.3 where low expression of *TALDO1 mRNA* was associated with favorable clinical outcome (*P* < 0.0001; Supplementary Fig. 3d).

The results from *mRNA* analysis encouraged us to further explore the potential value of TALDO1 protein levels using IHC in a cohort (*n* = 435) of luminal breast cancer. Representative images for staining of tumors classified as low and high are shown in (Supplementary Fig. 3e, f). TALDO1 expression was dichotomized into low and high using an H-score of 20. TALDO1 protein expression was associated with poor clinicopathological parameters including nodal stage (*P* = 0.009) and high Ki67 (*P* = 0.03).

To assess the clinical significance of TALDO1 expression in luminal breast cancer, we performed Kaplan–Meier analysis. Of interest, results demonstrated that high TALDO1 expression was associated with poor clinical outcome. Thus, patients whose tumors expressed high levels of TALDO1 experienced a higher risk of recurrence and death from breast cancer (*P* < 0.05; Fig. 4c, d) compared to patients with tumors in the TALDO1 low group who had a more favorable clinical outcome.

### TALDO1 expression associates with endocrine therapy failure

**Fig. 5:**
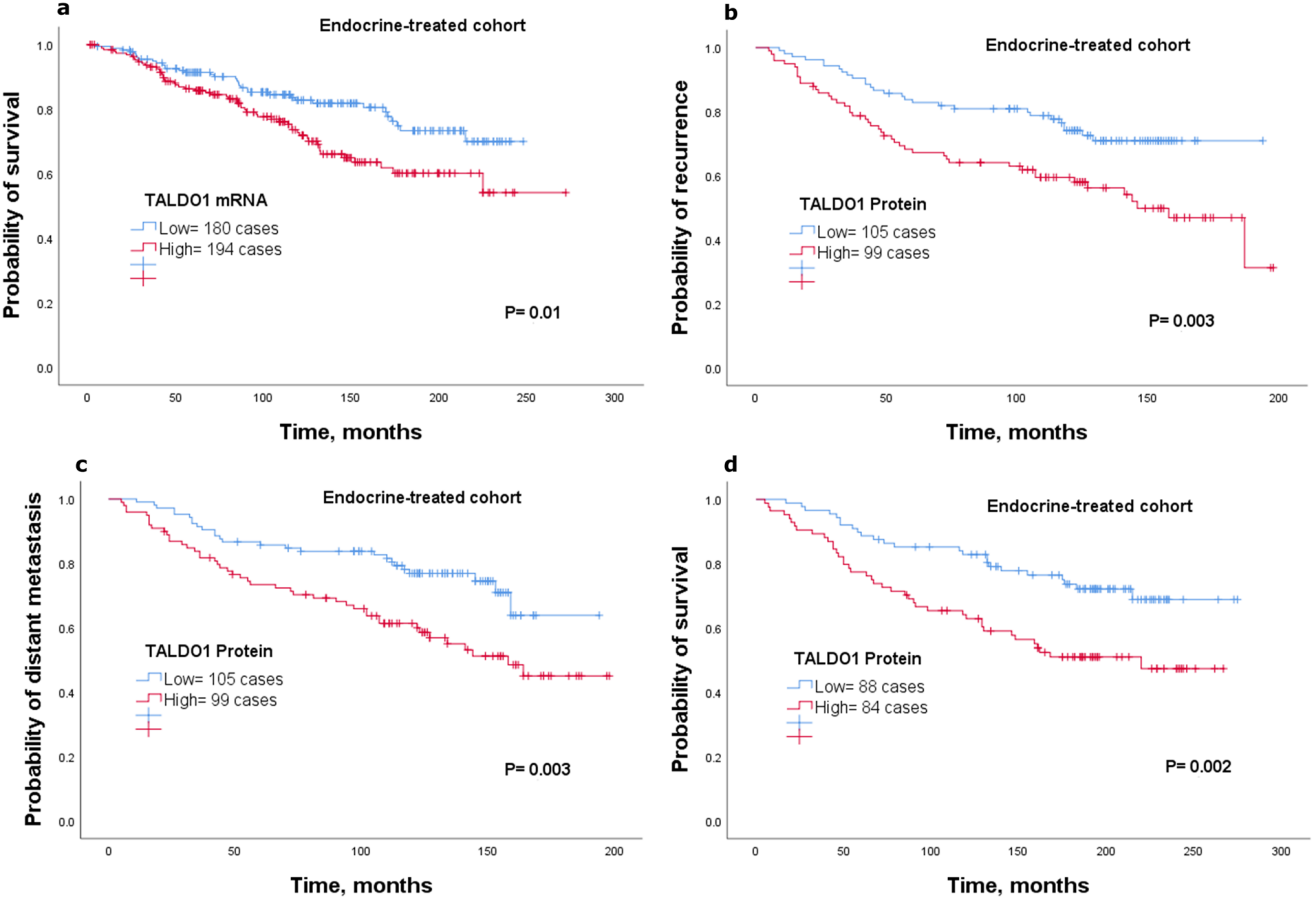
Kaplan–Meier of *TALDO1 mRNA* expression in patients with luminal breast cancer who received endocrine treatment only using the METABRIC cohort** a** survival. Kaplan–Meier of SLC1A5 protein expression in patients with luminal breast cancer who received endocrine treatment only using Nottingham cohort **b** recurrence**, c** distant metastasis, and **d** survival

To assess the clinical significance of TALDO1 expression in terms of response to endocrine therapy, we analyzed the clinical outcome of patients who received adjuvant endocrine treatment alone using *TALDO1 mRNA* and TALDO1 protein expression. Results from both *mRNA*, using the METABRIC cohort, and protein levels showed that patients who had high expression of *TALDO1 mRNA* or TALDO1 protein were associated with poor outcome compared to the low expression group (*P* < 0.05; Fig. 5a–d). Results of *mRNA* expression were validated using KM-Plotter dataset (*P* < 0.05; Supplementary Fig. 3 g–i). These findings suggest that TALDO1 is clinically associated with failure of endocrine treatment.

For independent prognostic value of TALDO1 in luminal breast cancer, multivariate analysis showed that protein expression of TALDO1 independently predicted poor clinical outcome (*P* < 0.05), (Supplementary Table 3). Further analysis on the subgroup of patients who were treated with endocrine therapy alone revealed that TALDO1 remains a predictive marker of high risk to recurrence, distant metastasis and risk of breast cancer death (*P* < 0.05), (Supplementary Table 3).

### SLC1A5/TALDO1 co-expression predicts poor clinical outcome after endocrine treatment

**Fig. 6:**
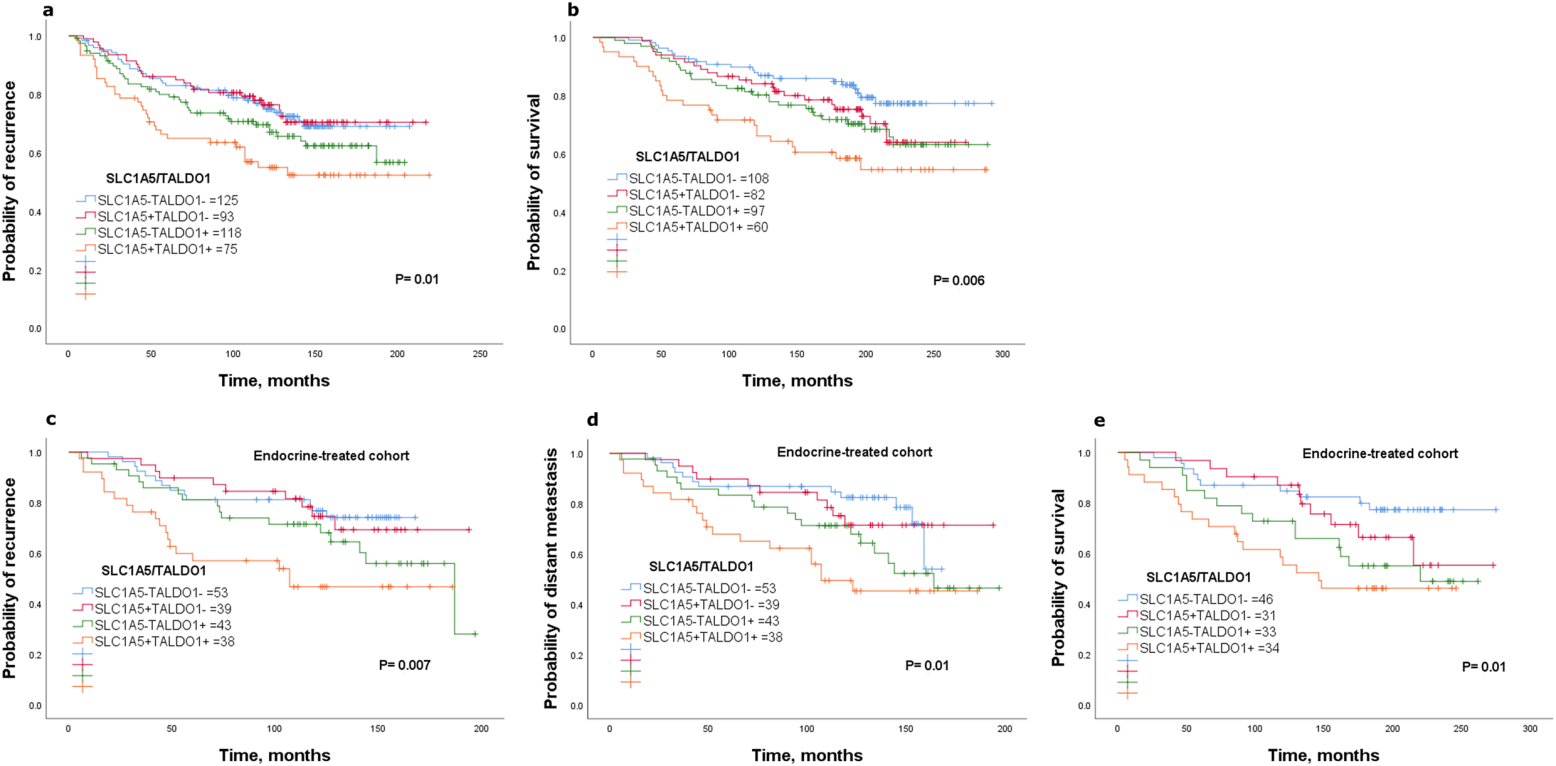
Kaplan–Meier of SLC1A5/TALDO1 protein co-expression as a prognostic marker for poor clinical outcome in luminal breast cancer** a** recurrence and** b** survival. Kaplan–Meier of SLC1A5/TALDO1 protein co-expression in patients with luminal breast cancer who received endocrine treatment only using Nottingham cohort **c** recurrence, **d** distant metastasis, and **e** survival

Next, we aimed to investigate whether co-expression of SLC1A5 and TALDO1 protein expression could also represent a marker of poor patient outcome and response to endocrine therapy. Patients were divided into four subgroups; SLC1A5-TALDO1- (both SLC1A5 and TALDO1 low expression), SLC1A5 + TALDO1- (high SLC1A5 and low TALDO1 expression), SLC1A5-TALDO1 + (low SLC1A5 and high TALDO1 expression) and SLC1A5 + TALDO1 + (both SLC1A5 and TALDO1 showing high expression). In luminal breast cancer, SLC1A5 + TALDO1 + co-expression was predictive of a high recurrence risk and death from breast cancer (*P* < 0.05; Fig. 6a, b). Next, we investigated their co-expression effect on the efficacy of endocrine treatment, by analyzing only those patients who received hormone therapy alone. Results showed that patients with SLC1A5 + TALDO1 + were more associated with unfavorable outcomes compared with the other subgroups (*P* < 0.05; Fig. 6c–e).

To investigate the prognostic ability of SLC1A5/TALDO1 co-expression independently of tumor size, grade and nodal stage, we performed multivariate Cox regression. In the whole cohort of luminal breast cancer, SLC1A5 + TALDO1 + was independently prognostic of a worse clinical outcome (*P* < 0.05), Supplementary Table 4. In those patients who were treated with only adjuvant endocrine treatment, SLC1A5 + TALDO1 + co-expression was independently predictive of high risk of recurrence, distant metastasis and shorter survival (*P* < 0.05), Supplementary Table 4. Taken together, the combined expression of SLC1A5 and TALDO1 has a utility value as a prognostic marker of poor clinical outcome in luminal breast cancer. Additionally, patients with luminal breast cancer could be classified either as having a good or poor response to endocrine therapy according to SLC1A5/TALDO1 co-expression.

### Association of SLC1A5 and TALDO1 expression with proliferation-related genes

As cellular proliferation a key mechanism that contributes to failure of response to endocrine treatment in breast cancer. We next investigated the correlation between the *mRNA* expression of *SLC1A5* and *TALDO1* and proliferation-related genes in patients with luminal breast cancer using METABRIC cohort. Results revealed a positive correlation between *SLC1A5* with the expression of *MKI67, CCNB1, CCNA2* and *CCND1* (*P* < 0.05), same results were found for *TALDO1* except *CCND1*, (Supplementary Table 5). These findings were validated using bc-GenExMiner v4.3 (*P* < 0.0001; Supplementary Fig. 4a–h).

## Discussion

Endocrine therapy has proven its enormous value in the treatment of luminal breast cancer. However, resistance to this type of treatment is a significant clinical issue for a large number of patients who will either not initially respond or will eventually relapse. Therefore, it is necessary to predict which patients will not potentially benefit from endocrine treatment through identifying valuable clinical biomarkers to guide the choice of treatment [22]. Amino acid transporters have an essential role in glutamine metabolism via transporting amino acids, glucose and other nutrients for driving tumor cell proliferation and survival. Despite the role of glutamine metabolism in promoting tumor progression being well documented [4], the potential roles of these transporters are less investigated particularly in terms of endocrine resistance in luminal breast cancer, apart from SLC38A2 and SLC7A5 [23, 24]. Here, we found a prognostic utility of the amino acid transporter SLC1A5 and the metabolic enzyme TALDO1 in luminal breast cancer and endocrine therapy.

High SLC1A5 expression has been recently reported in different types of cancer [7, 9, 12, 25], and identified as a prognostic marker of adverse outcome [25–27]. A recent study has shown that SLC1A5 is highly expressed in triple negative breast cancer [13]. In terms of luminal breast cancer previous study reported that low SLC1A5 expression and ER positivity correlated with good prognosis in 119 patients with breast cancer [28]. Consistent with that, we found high SLC1A5 expression, as a result of high transcription, is associated with aggressive clinicopathological features and high risk of recurrence in patients with luminal breast cancer. This finding suggests a critical role of this amino acid transporter in the progression and tumorigenesis of luminal breast cancer.

SLC1A5 has a key role in facilitating glutamine uptake and promotes glutamine metabolism [29]. Glutamine metabolism has also been linked to estrogen stimulation and it has been suggested as an essential mechanism for cell proliferation in luminal breast cancer cells [30]. A recent In vitro study demonstrated that SLC1A5 is upregulated in breast cancer cells resistant to endocrine therapy and that SLC1A5 inhibition decreased cell proliferation in aromatase inhibitors resistant breast cancer cells [31]. Consistent with those findings, we identified an association between SLC1A5 expression and endocrine therapy sensitivity in luminal breast cancer. Our results using clinical samples showed that patients with luminal breast cancer and high SLC1A5 expression are more likely to relapse after using endocrine therapy. In vitro, this finding was confirmed, where depletion of SLC1A5 in luminal breast cancer cells increased the sensitivity to tamoxifen treatment compared to control cells. Altogether, these findings suggest that SLC1A5 is an emerging key marker of endocrine therapy in luminal breast cancer.

Using co-expression networks analysis to find potential mechanisms with which SLC1A5 interacts in luminal breast cancer, we identified biological networks of 25 genes that correlated with SLC1A5. Subsequently, we sought to identify which were the most enriched molecular pathways and gene targets that are associated with SLC1A5 expression. The metabolic pathway and synthesis of amino acids were identified as the most enriched pathways. TALDO1 was enriched in these pathways and its expression was positively correlated with SLC1A5. TALDO1 is an enzyme that is involved in the pentose phosphate pathway, which is a key player in cellular metabolism and proliferation processes in cancer cells [32]. High expression of TALDO1 is found in different tumors including liver [33], and head/neck [34]. Additionally, increased expression of the pentose phosphate pathway enzymes, including TALDO1, accounts for metabolic reprogramming and contributes to cell proliferation in lung adenocarcinoma cells [35]. Consistent with these findings, in the present study, TALDO1 was co-expressed with SLC1A5 and increased expression of this metabolic gene contributed to the aggressive features of luminal breast cancer. Furthermore, our results revealed that high expression of *TALDO1 mRNA* and protein are significantly associated with poor outcomes in patients with luminal breast cancer. Taken together, these findings suggest that TALDO1 is a poor prognostic marker in patients with luminal breast cancer.

TALDO1 has previously been reported as a prognostic marker of poor response to HER2 inhibition in breast cancer patients [36]. However, no previous study has demonstrated the prognostic utility of TALDO1 in predicting the response to endocrine treatment in luminal breast cancer. Here, we have shown that patients with high TALDO1 expression have a poor outcome and greater risk of relapse after receiving endocrine therapy. This finding suggests that increased expression of TALDO1 is linked to endocrine resistance in luminal breast cancer, thus TALDO1 represents a dominant controller of metabolic flux within the pentose phosphate pathway. It might be that tamoxifen treatment affects cellular metabolism such TALDO1 which catalyze the rate-limiting step in the non-oxidative pentose phosphate pathway, which essential for cancer cells to maintain their highly proliferative state under tamoxifen treatment. However, the exact mechanism of how TALDO1 contributes to failure of endocrine therapy is unclear and needs further mechanistic investigations.

Proliferation is a key mechanism that contributes to failure of benefit from endocrine therapy in breast cancer. For instance, cyclin D1 is upregulated in a subgroup of patients with luminal breast cancer who do not respond to endocrine treatment [37]. A recent study showed that knockdown of SLC1A5 inhibits cell proliferation and arrests cell cycle in G0/G1 phase in gastric cancer [25]. In light of this, we found a positive correlation of *SLC1A5* and *TALDO1* expression with proliferation-associated genes, including *CCND1, CCNA2, CCNB1* and *MKI67*. Therefore, our finding suggests key roles for both *SLC1A5* and *TALDO1* in regulating cellular proliferation within the cell cycle of luminal breast cancer, which may initiate molecular mechanisms that affect cell response to endocrine therapy.

As our results demonstrated the prognostic value of SLC1A5 and TALDO1 singularly in luminal breast cancer, we also established the co-expression impact of these markers on the clinical outcome and efficacy of adjuvant endocrine treatment. Patients with SLC1A5 + TALDO1 + had the worst outcome and further analysis revealed a significant association between SLC1A5 + TALDO1 + expression and a high risk of recurrence and death from breast cancer in patients who had received adjuvant endocrine therapy only. These results further strengthen a possible role for SLC1A5 and TALDO1 in patients with luminal breast cancer and could be used to identify a subset of patients who endocrine therapy failure.

Even though our data demonstrate the prognostic and predictive value of SLC1A5 in luminal breast cancer, the exact mechanisms of how SLC1A5 expression contributes to endocrine resistance still require further mechanistic investigations. Such investigations should use tamoxifen resistant cells to demonstrate that SLC1A5 knockdown changes the sensitivity to tamoxifen, and to measure the intracellular glutamine levels in SLC1A5 knockdown resistant cells to give an insight into the mechanism of how SLC1A5 confers endocrine resistance. Additionally, apoptosis assay and cell cycle analysis to examine whether tamoxifen upon SLC1A5 knockdown induces cell death or cell cycle arrest are potential studies to be performed The predictive value of SLC1A5 on the benefit of endocrine therapy will also require further investigation in clinical trials of primary breast cancer treatment.

In conclusion, our findings suggested that the contribution of SLC1A5 expression in luminal breast cancer and failure of endocrine treatment might be through participation in the pentose phosphate pathway via regulation of TALDO1 expression essential for cancer cells growth and proliferation. Additionally, we suggest that SLC1A5 is a potential therapeutic target for overcoming endocrine resistance and demonstrates the prognostic value of both SLC1A5 and TALDO1 as biomarkers for predicting endocrine therapy guide in luminal breast cancer.

## Supplementary Information

Below is the link to the electronic supplementary material.

Supplementary file1 (PDF 1554 KB)

## Data Availability

The data that support the findings of this study are available from the corresponding author upon reasonable request.
