# Supplementary material for: SLC1A5 co-expression with TALDO1 associates with endocrine therapy failure in estrogen receptor-positive breast cancer
Source: Breast Cancer Res Treat. 2021 Jul 19;189(2):317–31. doi: 10.1007/s10549-021-06298-1 (PMC8357718; doi:10.1007/s10549-021-06298-1)
Supplement: Supplementary file 1 — Supplementary file1 (PDF 1554 KB) [file 10549_2021_6298_MOESM1_ESM.pdf]

Breast Cancer Research and Treatment

SLC1A5 co-expression with TALDO1 associates with endocrine therapy failure in oestrogen receptor-positive breast cancer

Lutfi H. Alfarsi,[1] Rokaya El Ansari[1], Madeleine L. Craze[1], Omar J. Mohammed[1], Brendah K. Masisi[1], Ian O. Ellis[1,2] , Emad A. Rakha[1,2] and Andrew R. Green[1*]

[1] Nottingham Breast Cancer Research Centre, Division of Cancer and Stem Cells, School of Medicine, University of Nottingham, University of Nottingham Biodiscovery Institute, University Park, Nottingham NG7 2RD;
[2] Cellular Pathology, Nottingham University Hospitals NHS Trust, Nottingham City Hospital, Hucknall Road, Nottingham NG5 1PB.

Correspondence:
Dr. Andrew R. Green. Nottingham Breast Cancer Research Centre, Division of Cancer and Stem Cells, School of Medicine, University of Nottingham Biodiscovery Institute, University Park, Nottingham NG7 2RD
Tel: (44) 115 8231407
Email: andrew.green@nottingham.ac.uk

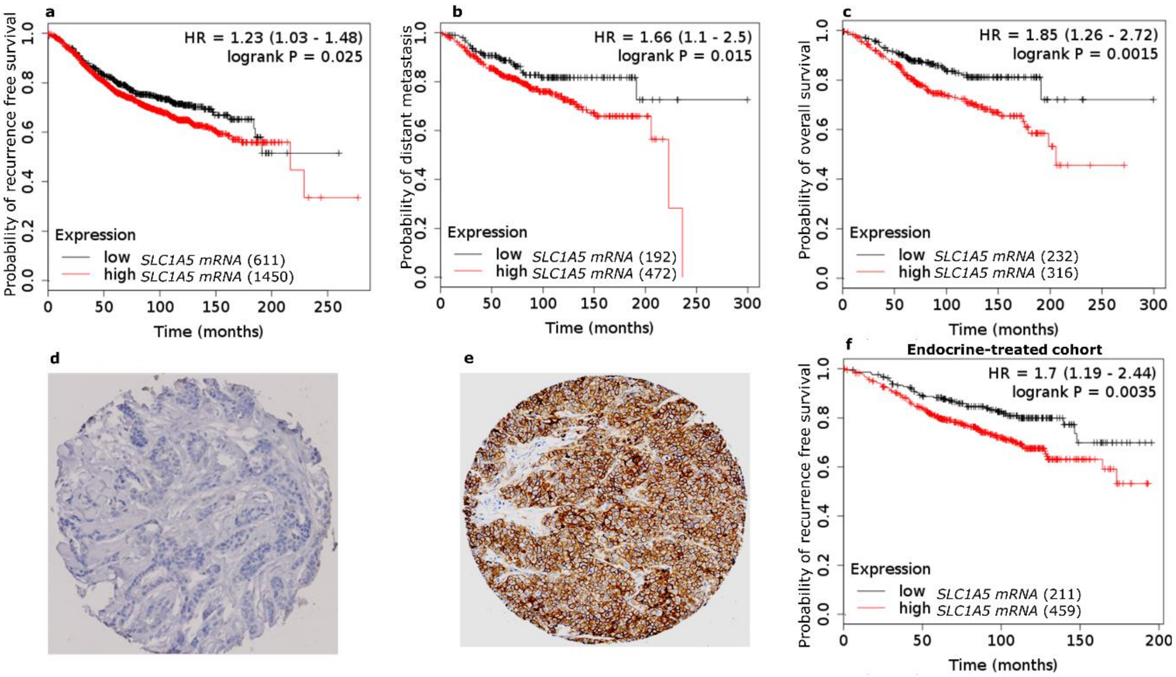

Supplementary figure 1: Kaplan–Meier of *SLC1A5 mRNA* and patient outcome in luminal breast cancer using KM-Plotter for **a)** recurrence free survival **b)** distant metastasis and **c)** overall survival. Representative immunostaining images of invasive breast cancer cores using IHC **d)** Negative and **e)** positive for SLC1A5 protein expression. Kaplan-Meier of *SLC1A5 mRNA* expression in patients with luminal breast cancer who received endocrine treatment only using KM-Plotter dataset **f)** recurrence free survival.

a

	Correlation Coefficient	*P*
TALDO1	0.23	< 0.0001
CYC1	0.21	< 0.0001
PYCR1	0.24	< 0.0001
TK1	0.24	< 0.0001
PPP5C	0.15	< 0.0001

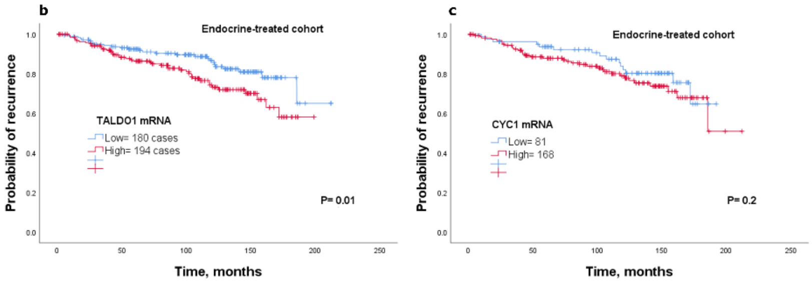

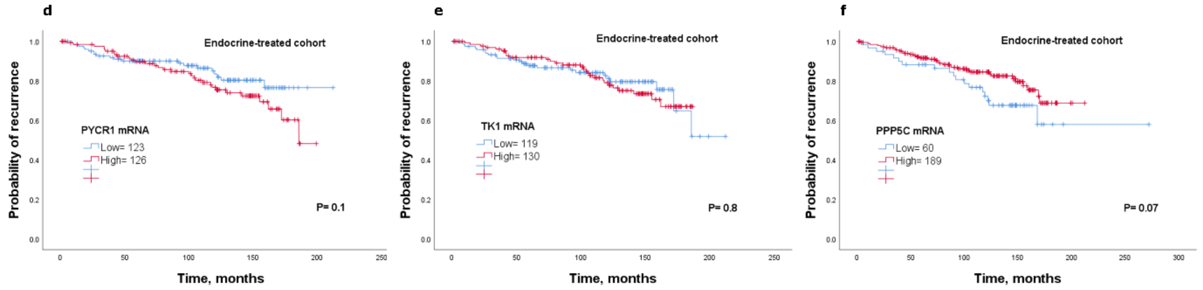

Supplementary figure 2: a) shows correlation of *TALDO1, CYC1, PYCR1, TK1* and *PPP5C* with *SLC1A5 mRNA* expression using bc-GenExMiner v4.3. Kaplan-Meier analysis and recurrence in patients with luminal breast cancer who received endocrine treatment only using METABRIC cohort for **b)** *TALDO1* **c)** *CYC1* **d)** *PYCR1* **e)** *TK1* and **f)** *PPP5C*.

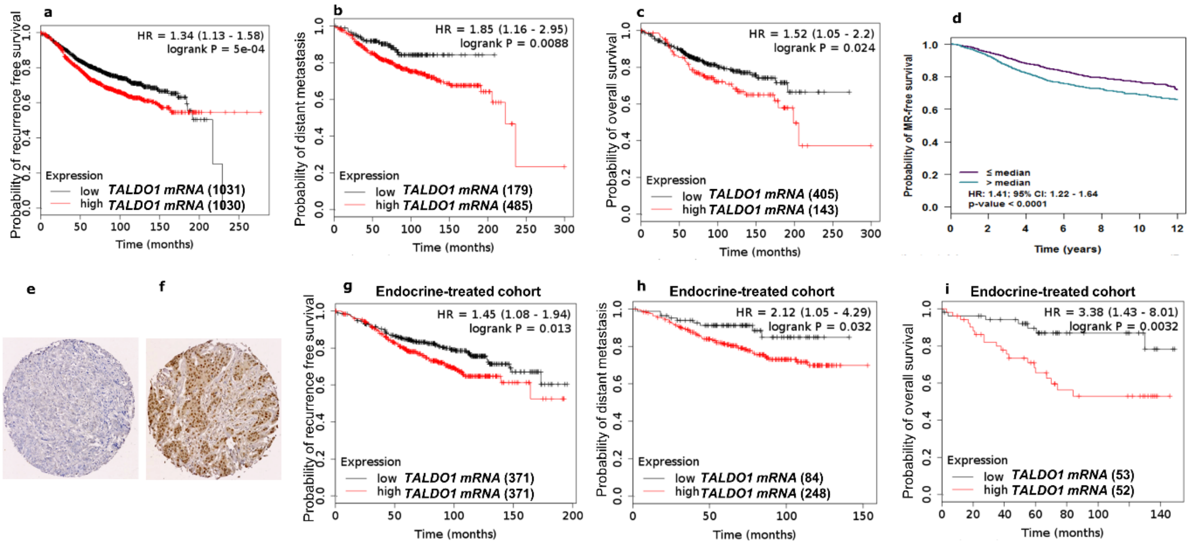

Supplementary figure 3: Kaplan–Meier of *TALDO1 mRNA* and patient outcome in luminal breast cancer using KM-Plotter dataset for **a)** recurrence free survival **b)** distant metastasis and **c)** overall survival, while **d)** Kaplan–Meier using bc-GenExMiner v4.3 for *TALDO1 mRNA* and survival. Representative immunostaining images of invasive breast cancer cores using IHC **e)** Negative and **f)** Positive for TALDO1 protein expression. Kaplan-Meier of *TALDO1 mRNA* expression in patients with luminal breast cancer who received endocrine treatment only using KM-Plotter **g)** recurrence free survival **h)** distant metastasis and **i)** overall survival.

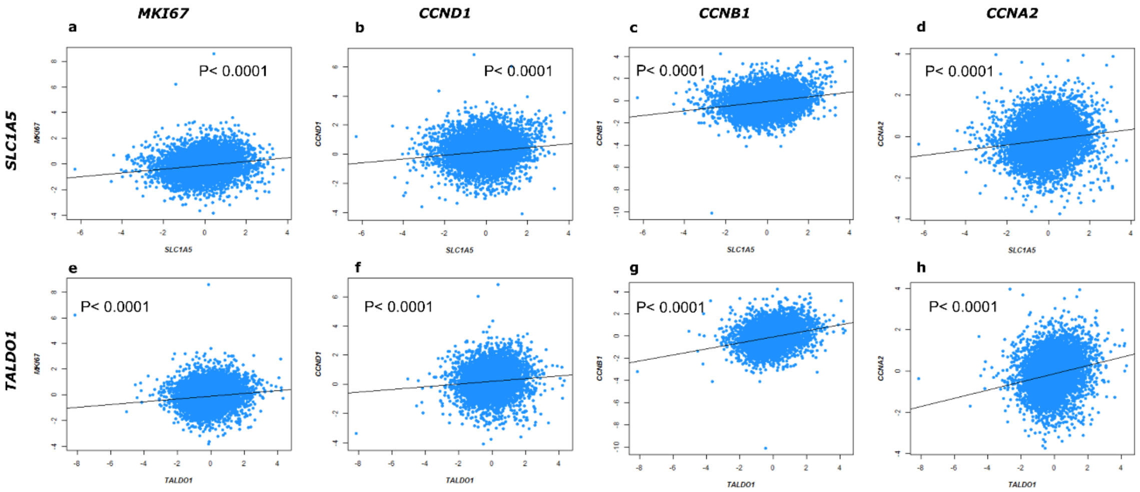

Supplementary figure 4: *SLC1A5 mRNA* correlation with proliferation associated-genes including **a)** *MKI67* **b)** *CCND1* **c)** *CCNB1* and **d)** *CCNA2*, and *TALDO1 mRNA* correlation with **e)** *MKI67* **f)** *CCND1* **g)** *CCNB1* and **h)** *CCNA2* using bc-GenExMiner v4.3 dataset.

Supplementary table 1: Clinicopathological characteristics of luminal breast cancer cohorts.

**Parameters**	**METABRIC cohort** ***mRNA***	**Nottingham cohort Protein**
	No. (%)	No. (%)
**Age**		
<50	228 (15)	449 (31)
≥50	1278 (85)	1006 (69)
**Tumour size (cm)**		
<2 cm	475 (31.5)	806 (55.7)
≥2 cm	1031 (68.5)	640 (44.3)
**Tumour grade**		
1	166 (11.5)	388 (24.7)
2	707 (49.1)	661 (42.1)
3	565 (38.4)	522 (33.2)
**Nottingham Prognostic Index**		
GPG	623 (41.3)	598 (41.4)
MPG	772 (51.2)	668 (46.1)
PPG	111 (7.5)	180 (12.5)
**Nodal stage**		
1	404 (36.2)	1025 (65.1)
2	634 (56.8)	439 (27.9)
3	78 (7)	111 (7)
**Vascular invasion**		
negative	N/A	1000 (69)
positive		439 (31)
**Endocrine therapy alone**		
No	234 (15.5)	810 (50)
Yes	384 (25.5)	554 (35)
Other*	888 (59)	237 (15)
**Recurrence**		
No	496 (33)	953 (60)
Yes	118 (7)	618 (38)
Unknown	892 (60)	30 (2)
**Distant metastasis**		
No	476 (31)	1079 (67)
Yes	143 (10)	514 (32)
Unknown	887 (59)	8 (1)
**Progesterone receptor**		
Negative	486 (23.2)	300 (21.3)
Positive	1020 (76.8)	1103 (78.7)

GPG: Good prognostic group**; MPG:** Moderate prognostic group**; PPG:** Poor prognostic group

***:** Include patients who received chemotherapy alone or combination of chemotherapy and endocrine therapy.

Supplementary table 2: Multivariate cox analysis of associations between SLC1A5 protein expression and clinicopathological parameters using Nottingham cohort.

**Luminal breast cancer cohort**			
	**Distant metastasis free survival**		
	HR (95% CI)	***P***	***P****
SLC1A5	1.2 (1.0-1.5)	0.028	0.03
Tumor size	1.5 (1.2-1.8)	0.00005	0.0001
Tumor grade	1.5 (1.3-1.8)	1.7e-10	< 0.0001
Nodal stage	1.8 (1.5-2.1)	7.9e-17	< 0.0001
	**Breast cancer specific survival**		
	HR (95% CI)	***P***	***P****
SLC1A5	1.3 (1.0-1.6)	0.01	0.012
Tumor size	1.4 (1.1-1.8)	0.0003	0.0005
Tumor grade	1.6 (1.4-1.9)	2.2e-11	< 0.0001
Nodal stage	1.6 (1.4-1.8)	1.7e-10	< 0.0001
**Endocrine-treated cohort**			
	**Breast cancer specific survival**		
	HR (95% CI)	***P***	***P****
SLC1A5	1.4 (1.0-1.9)	0.029	0.03
Tumor size	1.4 (1.0-2.0)	0.026	0.04
Tumor grade	2.2 (1.6-3.1)	1.5e-7	< 0.0001
Nodal stage	1.9 (1.5-2.4)	1.3e-7	< 0.0001

P*: Adjusted p-value

Supplementary table 3: Multivariate cox analysis of associations between TALDO1 protein expression and clinicopathological parameters using Nottingham cohort.

**Luminal breast cancer cohort**			
	**Recurrence free survival**		
	HR (95% CI)	***P***	***P****
TALDO1	1.5 (1.1-2.1)	0.006	0.01
Tumor size	1.5 (1.0-2.1)	0.013	0.02
Tumor grade	1.2 (1.0-1.6)	0.033	0.04
Nodal stage	1.5 (1.2-1.9)	0.0002	0.001
	**Distant metastasis free survival**		
	HR (95% CI)	***P***	***P****
TALDO1	1.5 (1.1-2.1)	0.009	0.01
Tumor size	2.0 (1.4-2.9)	0.00003	0.0002
Tumor grade	1.3 (1.0-1.7)	0.01	0.012
Nodal stage	1.5 (1.2-2.0)	0.0001	0.0003
	**Breast cancer specific survival**		
	HR (95% CI)	***P***	***P****
TALDO1	1.8 (1.2-2.5)	0.001	0.0013
Tumor size	2.0 (1.3-3.0)	0.00042	0.001
Tumor grade	1.6 (1.2-2.2)	0.00046	0.008
Nodal stage	1.7 (1.3-2.3)	0.00001	0.0001

**Endocrine-treated cohort**			
	**Recurrence free survival**		
	HR (95% CI)	***P***	***P****
TALDO1	2.2 (1.3-3.6)	0.001	0.002
Tumor size	1.6 (1.0-2.7)	0.03	0.05
Tumor grade	1.5 (0.9-2.2)	0.05	0.06
Nodal stage	1.7 (1.2-2.3)	0.001	0.005
	**Distant metastasis free survival**		
	HR (95% CI)	***P***	***P****
TALDO1	2.3 (1.4-3.9)	0.001	0.002
Tumor size	1.9 (1.1-3.3)	0.01	0.015
Tumor grade	1.7 (1.1-2.7)	0.012	0.016
Nodal stage	1.7 (1.2-2.3)	0.001	0.005
	**Breast cancer specific survival**		
	HR (95% CI)	***P***	***P****
TALDO1	2.4 (1.4-4.1)	0.001	0.002
Tumor size	2.0 (1.1-3.5)	0.013	0.016
Tumor grade	2.2 (1.3-3.6)	0.001	0.002
Nodal stage	1.7 (1.2-2.4)	0.002	0.005

P*: Adjusted p-value

Supplementary table 4: Multivariate cox analysis of associations between SLC1A5/TALDO1 protein co-expression and clinicopathological parameters using Nottingham cohort.

**Luminal breast cancer cohort**			
	**Recurrence free survival**		
	HR (95% CI)	***P***	***P****
SLC1A5+TALDO1+ vs SLC1A5-TALDO1-	1.5 (1.0-2.4)	0.03	0.05
Tumor size	1.5 (1.1-2.1)	0.008	0.02
Tumor grade	1.2 (1.0-1.6)	0.04	0.05
Nodal stage	1.5 (1.1-1.9)	0.001	0.005
	**Distant metastasis free survival**		
	HR (95% CI)	***P***	***P****
SLC1A5+TALDO1+ vs SLC1A5-TALDO1-	1.7 (1.0-2.7)	0.02	0.025
Tumor size	2.1 (1.5-3.1)	0.00002	0.0001
Tumor grade	1.3 (1.0-1.7)	0.01	0.016
Nodal stage	1.5 (1.2-1.9)	0.0003	0.0008
	**Breast cancer specific survival**		
	HR (95% CI)	***P***	***P****
SLC1A5+TALDO1+ vs SLC1A5-TALDO1-	2.1 (1.2-3.6)	0.005	0.006
Tumor size	2.0 (1.3-3.0)	0.0004	0.001
Tumor grade	1.6 (1.2-2.2)	0.002	0.003
Nodal stage	1.7 (1.3-2.2)	0.00004	0.0002
**Endocrine-treated cohort**			
	**Recurrence free survival**		
	HR (95% CI)	***P***	***P****
SLC1A5+TALDO1+ vs SLC1A5-TALDO1-	2.5 (1.3-4.7)	0.005	0.01
Tumor size	1.6 (1.0-2.7)	0.04	0.06
Tumor grade	1.4 (0.9-2.1)	0.1	0.12
Nodal stage	1.6 (1.1-2.3)	0.003	0.01
	**Distant metastasis free survival**		
	HR (95% CI)	***P***	***P****
SLC1A5+TALDO1+ vs SLC1A5-TALDO1-	2.5 (1.3-4.9)	0.006	0.01
Tumor size	1.9 (1.1-3.2)	0.01	0.016
Tumor grade	1.6 (1.0-2.6)	0.03	0.037
Nodal stage	1.6 (1.1-2.3)	0.004	0.02
	**Breast cancer specific survival**		
	HR (95% CI)	***P***	***P****
SLC1A5+TALDO1+ vs SLC1A5-TALDO1-	2.6 (1.3-5.4)	0.007	0.01
Tumor size	1.9 (1.1-3.3)	0.02	0.025
Tumor grade	2.0 (1.2-3.4)	0.005	0.01
Nodal stage	1.6 (1.1-2.4)	0.005	0.02

P*: Adjusted p-value

Supplementary table 5: Correlation of *SLC1A5* and *TALDO1 mRNA* expression with the proliferation related-genes in luminal breast cancer using METABRIC cohort.

	SLC1A5 mRNA		
	Correlation Coefficient	***P***	***P****
MKI67	0.21	7.89e-18	< 0.0001
CCNB1	0.25	6.21e-24	< 0.0001
CCNA2	0.19	1.01e-13	< 0.0001
CCND1	0.17	6.80e-12	< 0.0001
	TALDO1 mRNA		
	Correlation Coefficient	P	P*
MKI67	0.12	5.93e-7	< 0.0001
CCNB1	0.08	0.002	0.0027
CCNA2	0.33	1.91e-40	< 0.0001
CCND1	-0.004	0.89	3.56

P*: Adjusted p-value
